# The Rice High-Affinity K^+^ Transporter OsHKT2;4 Mediates Mg^2+^ Homeostasis under High-Mg^2+^ Conditions in Transgenic *Arabidopsis*

**DOI:** 10.3389/fpls.2017.01823

**Published:** 2017-10-24

**Authors:** Chi Zhang, Hejuan Li, Jiayuan Wang, Bin Zhang, Wei Wang, Hongxuan Lin, Sheng Luan, Jiping Gao, Wenzhi Lan

**Affiliations:** ^1^State Key Laboratory for Pharmaceutical Biotechnology, NJU–NFU Joint Institute for Plant Molecular Biology, College of Life Sciences, Nanjing University, Nanjing, China; ^2^National Key Laboratory of Plant Molecular Genetics, Institute of Plant Physiology and Ecology, Shanghai Institutes for Biological Sciences, Chinese Academy of Sciences, Shanghai, China; ^3^Department of Plant and Microbial Biology, University of California, Berkeley, Berkeley, CA, United States

**Keywords:** *Arabidopsis*, HKT transporter, MGT transporter, Mg^2+^ permeable, rice

## Abstract

Rice (*Oryza sativa*; background Nipponbare) contains nine *HKT* (high-affinity K^+^ transport)-like genes encoding membrane proteins belonging to the superfamily of Ktr/TRK/HKT. OsHKTs have been proposed to include four selectivity filter-pore-forming domains homologous to the bacterial K^+^ channel KcsA, and are separated into OsHKT1s with Na^+^-selective activity and OsHKT2s with Na^+^-K^+^ symport activity. As a member of the OsHKT2 subfamily, OsHKT2;4 renders Mg^2+^ and Ca^2+^ permeability for yeast cells and *Xenopus laevis* oocytes, besides K^+^ and Na^+^. However, physiological functions related to Mg^2+^ *in planta* have not yet been identified. Here we report that OsHKT2;4 from rice (*O. sativa*; background Nipponbare) functions as a low-affinity Mg^2+^ transporter to mediate Mg^2+^ homeostasis in plants under high-Mg^2+^ environments. Using the functional complementation assay in Mg^2+^-uptake deficient *Salmonella typhimurium* strains MM281 and electrophysiological analysis in *X. laevis* oocytes, we found that OsHKT2;4 could rescue the growth of MM281 in Mg^2+^-deficient conditions and induced the Mg^2+^ currents in oocytes at millimolar range of Mg^2+^. Additionally, overexpression of OsHKT2;4 to *Arabidopsis* mutant lines with a knockout of *AtMGT6*, a gene encoding the transporter protein necessary for Mg^2+^ adaptation in *Arabidopsis*, caused the Mg^2+^ toxicity to the leaves under the high-Mg^2+^ stress, but not under low-Mg^2+^ environments. Moreover, this Mg^2+^ toxicity symptom resulted from the excessive Mg^2+^ translocation from roots to shoots, and was relieved by the increase in supplemental Ca^2+^. Together, our results demonstrated that OsHKT2;4 is a low-affinity Mg^2+^ transporter responsible for Mg^2+^ transport to aerials in plants under high-Mg^2+^ conditions.

## Introduction

Apart from atmospheric oxygen and soil-derived water, plants require a range of minerals for their growth and development. As two major essential mineral nutrients for plant growth, K and Mg are available to plants in the ionic form (K^+^ and Mg^2+^), and are transported into root cells by the plasma membrane-localized channels and transporters. Up to now, most studies are focused on identifying the active, high-affinity channels and transporters, which function in K^+^ and Mg^2+^ uptake from the nutrient-deficient environments (Hirsch et al., 1998; Li et al., 2001; Chérel et al., 2014; Mao et al., 2014). However, large majority of channels and transporters necessary for plants adaptation to nutrient-enriched conditions remain unknown.

Due to its key role in salt tolerance, high-affinity K^+^ transporters (HKTs) family has been widely studied and most of its members are characterized as being permeable for specific ions in heterologous expression systems (Uozumi et al., 2000; Horie et al., 2001; Mäser et al., 2002b; Garciadeblas et al., 2003; Yao et al., 2010). HKTs in plants and their K^+^ transporter (Trk and Ktr) counterparts in fungi and bacteria form a HKT/Trk/Ktr superfamily (Rodriguez-Navarro, 2000; Corratgé-Faillie et al., 2010; Yamaguchi et al., 2013). Plant HKT transporters are divided into two subgroups based on phylogenetic analyses to date (Mäser et al., 2002a; Platten et al., 2006; Horie et al., 2009; Hauser and Horie, 2010). Group I HKT members (HKT1s) are associated with retrieval of Na^+^ from xylem in root or sheath restricting transport and accumulation of salt in sensitive leaf tissues (Davenport et al., 2007; Munns and Tester, 2008). Grass species evolved a second class of HKT proteins, and comprehensive analysis of this group II HKTs (HKT2s) has been made in rice (*Oryza sativa L.*) with up to four members, OsHKT2;1, OsHKT2;2, OsHKT2;3, and OsHKT2;4 characterized for the structure, expression, and function (Ariyarathna et al., 2016). Most of HKT2s members function as Na^+^/K^+^ transporters with a role in maintaining Na^+^/K^+^ homeostasis in plants (Horie et al., 2007, 2011; Lan et al., 2010; Yao et al., 2010; Nieves-Cordones et al., 2016). OsHKT2;4 seems to be an exception as it exhibited permeability to a wide range of cations, including Ca^2+^ and Mg^2+^ when it was expressed in *Xenopus laevis* oocytes (Lan et al., 2010; Horie et al., 2011). However, its physiological function in rice is still unknown.

The *Arabidopsis* genome contains a single *HKT* homolog, *AtHKT1;1*, which functions as a Na^+^-selective uniporter and is not permeable to Ca^2+^ and Mg^2+^ (Davenport et al., 2007; Møller et al., 2009; Lan et al., 2010), suggesting that there are alternative transporters responsible for Ca^2+^ and Mg^2+^ transport in *Arabidopsis*. Ca^2+^ and Mg^2+^ are two of the most abundant divalent cations in living plant cells. Ca^2+^ is utilized to strengthen cell walls and a versatile messenger in almost all physiological processes in plants (Tang and Luan, 2017). The prominent role of Mg^2+^ is as the central atom of the chlorophyll molecule (Larkin, 2016), and it also participates in cation balance and activation of various enzymes in many fundamental processes (Shaul, 2002; Knoop et al., 2005; Bose et al., 2013). Although Ca^2+^ and Mg^2+^ are essential macronutrients required for plant growth, their overdose in the environment is toxic to plants (Tang et al., 2015; Oda et al., 2016). Thus, the transporters responsible for Ca^2+^ and Mg^2+^ homeostasis is of great importance for plant survival under low or high Ca^2+^ and Mg^2+^ conditions (Miedema et al., 2001; Li et al., 2008; Hermans et al., 2013; Mao et al., 2014; Oda et al., 2016). In contrast to the ambiguous research in Ca^2+^ transport, a family of Mg^2+^ transporters in *Arabidopsis* named as AtMGT (Li et al., 2001) or AtMRS2 (Gebert et al., 2009) has been studied extensively, and is found to play pivotal roles in Mg^2+^ transport and homeostasis in *Arabidopsis*. One of its members, AtMGT6/MRS2-4, is a high-affinity Mg^2+^ transporter, and loss-of-function of AtMGT6/MRS2-4 caused the severe growth retardation of *Arabidopsis* plants under low-Mg^2+^ conditions (Mao et al., 2014). Interestingly, AtMGT6/MRS2-4 also confers plants adaptation to high-Mg^2+^ conditions (Oda et al., 2016). Thus, *atmgt6* plant with loss-of-function of AtMGT6/MRS2-4 displays the deficient Mg^2+^ transport under wide range of Mg^2+^ concentrations, and is a promising expression system to examine whether the potential transporters possess physiological functions relevant to Mg^2+^ in plants.

Although OsHKT2;4 was demonstrated to be permeable for Mg^2+^ in *X. laevis* oocytes (Lan et al., 2010; Horie et al., 2011), this has been challenged in an independent study (Sassi et al., 2012). Here, we applied the *Salmonella typhimurium* MM281, a bacteria mutant lacking Mg^2+^ transport capacity useful for identifying the Mg^2+^ transport activities of potential transporters (Li et al., 2001; Gebert et al., 2009), to analyze the possible Mg^2+^ transport through OsHKT2;4. Furthermore, its function on Mg^2+^ homeostasis was also explored in *X. laevis* oocytes and transgenic *atmgt6 Arabidopsis* lines. Our results revealed that OsHKT2;4 is an effective Mg^2+^ transporter in maintaining Mg^2+^ homeostasis, probably through functional coordination with MGT-type transporters *in planta*.

## Materials and Methods

### Plant Materials and Growth Conditions

*Arabidopsis thaliana* Columbia (Col-0) ecotype was used in this study. The T-DNA insertion mutant *atmgt6* (SALK_203866) was obtained from the *Arabidopsis* Biological Resource Center. Homozygous individuals of *atmgt6* were screened by PCR using primers listed in Supplementary Table 1. For on-plate growth assays, seeds were sterilized with 75% ethanol for 2 min, washed three times, and sown on half-strength Murashige and Skoog (MS) medium containing 0.75 mM Mg^2+^, 1.5 mM Ca^2+^, 1% sucrose (Sigma) and solidified with 0.8% phytoblend (Caisson Labs). The plates were kept at 4°C for 2 days and then were placed vertically in growth chamber under 90 μmol⋅m^-2^⋅s^-1^ light intensity with a 16 h light/8 h dark photoperiod. Three-day-old seedlings were transferred onto media containing various ions as indicated in the figure legends. For hydroponic cultures, 7-day-old seedlings germinated in half-strength MS (1/2 MS) were transferred to one-sixth-strength (1/6 MS) hydroponic medium containing 0.25 mM Mg^2+^ and 0.5 mM Ca^2+^ without sucrose for another 7 days. Plants were then transferred to hydroponic 1/6 MS media containing various contents of Mg^2+^. Plant materials were harvested for further analyses 2 days after treatment.

### Functional Complementation of Mg^2+^-Transport by *Salmonella typhimurium* Mutant Strain MM281

The *S. typhimurium* mutant MM281, which lacks the Mg^2+^ transporter-*CorA*, *MgtA*, and *MgtB*, is used as a system for functional complementation analysis of candidate Mg^2+^-transporter genes. MM281 competent cells were transformed with empty pTrc99A vector, *AtMGT10-*pTrc99A or *OsHKT2;4-*pTrc99A plasmid by electroporation. Cells were plated onto LB medium containing 10 mM Mg^2+^ and indicated antibiotics (34 μg⋅mL^-1^ chloramphenicol and 100 μg⋅mL^-1^ ampicillin), and incubated at 37°C overnight. The transformants were confirmed by PCR amplification and individual positive ones were grown in liquid LB medium containing 10 mM Mg^2+^ and antibiotics as indicated above. Fifty micrometer IPTG was applied for the induction of protein expression. The liquid cultures were adjusted to OD_600_ = 1.0, diluted in a 10-fold series, and spotted 3 μL onto N-minimal medium supplemented with different concentrations of MgSO_4_ and the antibiotics. Growth of different strains was pictured after incubation at 37°C for 2 days. The growth rate of the three strains in liquid medium was also monitored as previously described (Mao et al., 2014). After growing in liquid LB medium to OD_600_ of 0.6–0.8, cells were harvested by centrifugation at 5000 × *g* for 10 min, washed twice with distilled water to remove excess Mg^2+^, and resuspended in distilled water. N-minimal medium was prepared with various concentrations of MgSO_4_ (0.1, 0.5, 1, and 10 mM). Cells were then adjusted to a final OD_600_ of 0.001–0.002. The growth of the cultures was monitored and was plotted as a function of growth time.

### Plasmid Construction and Plant Transformation

For the constructs used in functional complementation assay in MM281 strain, the *OsHKT2;4* cDNA fragment was amplified using the primers *OsHKT2;4*-FC and *OsHKT2;4*-RC and ligated to the pTrc99A vector. For overexpressing *OsHKT2;4* in wild type and the *atmgt6* mutant, the genomic fragment (containing a 1.92 kb promoter region upstream of the *ATG* starting codon and 1731 bp coding region of *OsHKT2;4*) was amplified using primer pair *OsHKT2;4*-OE-F and *OsHKT2;4*-OE-R and cloned into pCAMBIA1300 vector. This construct was introduced into *Agrobacterium tumefaciens* strain GV3101 by electroporation and was selected on 1/2 MS medium containing kanamycin. The selected positive transformant was used to transform developing floral tissues of 4-week-old *atmgt6* plants using the flora dip method (Clough and Bent, 1998). For expression in *X. laevis* oocytes, *OsHKT2;4* cDNA was cloned into the pGEMHE vector downstream from the T7 promoter using primers *OsHKT2;4*-FP and *OsHKT2;4*-RP. All primer pairs were listed in Supplementary Table 1.

### Gene Expression Analysis

Total RNA was extracted from rosette leaves using the TRizol Reagent (Invitrogen), and the first-strand cDNA was synthesized by M-MLV Reverse Transcriptase (Promega) following the manufacturer’s instructions. The semi-quantitative RT-PCR analysis of gene expression using cDNA of Col-0, *atmgt6*, OE29, and OE24 followed by a 26 cycles of PCR amplification. *AtActin2* (AT3G18780) was used as the internal reference. Primers used are listed in the Supplementary Table 1.

### Expression in *Xenopus laevis* Oocytes and Two-Electrode Voltage Clamp

cRNA was synthesized from 1 μg linearized DNA template using a mMessage mMachine *in vitro* transcription kit (Ambion) according to the manufacturer’s recommendations and stored at -80°C. Stage V to VI *X. laevis* oocytes were harvested, defolliculated, and cultured in ND96 solution containing 96 mM NaCl, 2 mM KCl, 1.8 mM CaCl_2_, 1 mM MgCl_2_, 25 μg⋅mL^-1^ gentamicin, pH 7.4 adjusted with 5 mM HEPES/NaOH. Approximately 50 ng of cRNA, in a total volume of 23 nL, was injected into each *X. laevis* oocyte. Oocytes of 2 days after injection were used for two-electrode voltage-clamp analysis. The perfusion solution was used as described previously (Lan et al., 2010) with some modifications. The perfusion solution contained (in mM) 1 K-gluconate, 1 Na-gluconate, 185 mannitol, and 10 Mes-Tris (pH 7.4). The recording pipette contained 3 M KCl. The currents were recorded by hyperpolarized pulses of a 0.2 s prepulse at -40 mV followed by voltage steps of 60 to -150 mV (in 15 mV decrements, 1.8 s duration) followed by a 1.5 s deactivation at 0 mV. The current-voltage (*I-V*) curves plot current values at the end of each voltage-clamp episode (*t* = 2 s, *n* = 6 for each group).

### Ion Content Measurement

Two-week-old hydroponically grown plants were exposed to solution containing different concentrations of Mg^2+^. After 2-day exposure, both the roots and shoots were harvested and sampled for analysis. The dry weight (DW) of the samples was measured after drying for 48 h at 60°C. Subsequently, the samples were digested in 0.5 ml of 70% HNO_3_ at 100°C for 30 min on a digester (DigiBlock ED16, LabTech). Ion concentration was measured by Inductively Coupled Plasma-Mass Spectrometry (ICP-MS) (PerkinElmer NexION 300).

## Results

### OsHKT2;4 Rescued the Growth of Bacterial Strain MM281 in the Mg^2+^-Deficient Medium

To determine whether OsHKT2;4 functions in Mg^2+^ transport, a cDNA fragment containing the complete open reading frame with 1530 bases encoded a protein of 509 residues was cloned and expressed in *S. typhimurium* mutant strain MM281. MM281 is incapable of loading Mg^2+^ into cellular compartment, as it lacks three functional Mg^2+^ transporters *CorA*, *MgtA*, and *MgtB*, and its growth is retarded or arrested when the culture medium contains less than 10 mM Mg^2+^ (Townsend et al., 1995; Li et al., 2001). Therefore, complementation of this strain has proved useful in identifying and developing information about potential Mg^2+^ transporters, including AtMGTs (Li et al., 2001, 2008; Hicks et al., 2003; Mao et al., 2008; Chen et al., 2009; Mao et al., 2014).

**FIGURE 1:**
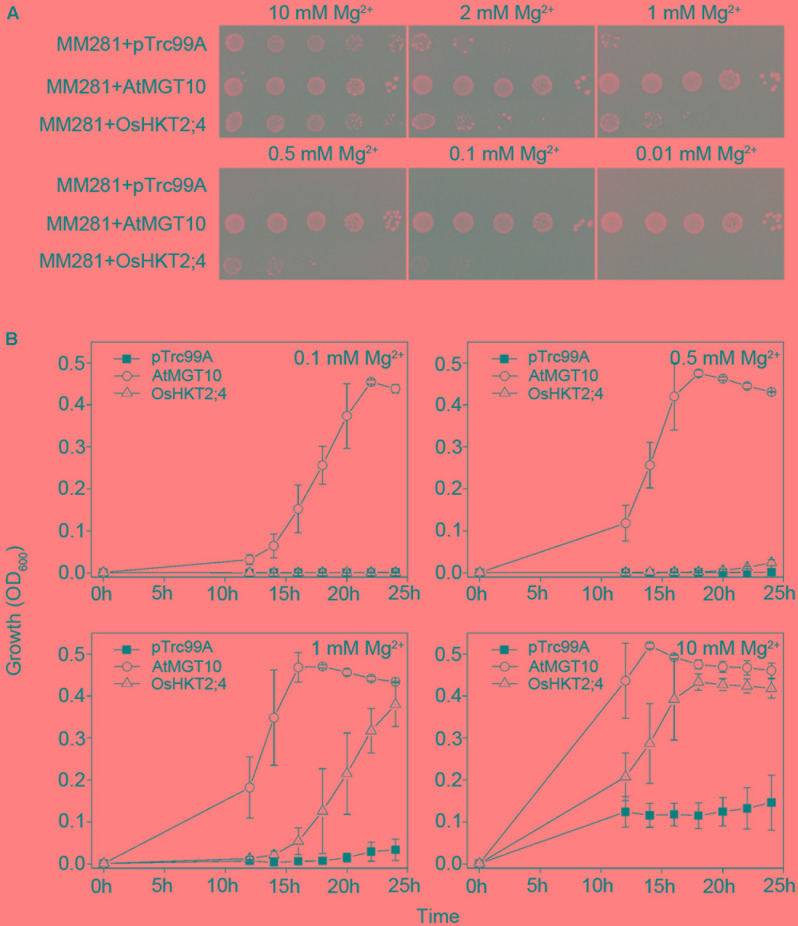
Complementation of growth defects of bacterial mutant strains MM281 by OsHKT2;4 under the low-Mg^2+^ conditions. **(A)** Growth of bacterial strains on N-minimal medium containing 0.01, 0.1, 0.5, 1, 2, or 10 mM Mg^2+^. The strains used in this assay were the strains MM281 transformed with the empty pTrc99A vector only (MM281+pTrc99A), coding sequence of *MGT10* in the pTrc99A vector (MM281+MGT10), or coding sequence of *OsHKT2;4* in pTrc99A vector (MM281+OsHKT2;4). From left to right is a 10-fold dilution series of bacterial cultures. **(B)** Growth curves of bacterial strains in liquid cultures. Bacterial cells described in **(A)** were grown in N-minimal liquid medium containing increasing concentrations of Mg^2+^ from 0.1 to 10 mM. Aliquots of the cultures were taken and monitored every 2 h by OD_600_ readings for the cell density from 10 to 24 h. Data are represented as the mean ± SD, *n* = 3.

We used *AtMGT10* (AT5G22830), also named as *AtMRS2-11*, as the positive control in the complementation assay of MM281 due to its high affinity in Mg^2+^ transport in MM281 system (Li et al., 2001). As shown in **Figure 1A**, the MM281 mutant strains exogenously expressing empty vector pTrc99A, *AtMGT10*-pTrc99A, or *OsHKT2;4*-pTrc99A grew normally in the medium with 10 mM Mg^2+^. The strains expressing *OsHKT2;4*-pTrc99A exhibited faster growth than those expressing the empty pTrc99A vector in the media containing low concentrations of Mg^2+^ (1 and 2 mM), and still grew but to a less extent in the media containing 500 and 100 μM Mg^2+^, while the control did not grow at these conditions (**Figure 1A**), suggesting that OsHKT2;4 renders the mutant strains more tolerant to Mg^2+^ deficiency by enhancing the Mg^2+^ transport activity. However, OsHKT2;4 was less effective to restore the growth of mutant strains compared with AtMGT10 in the media containing insufficient Mg^2+^. For example, AtMGT10 rescued MM281 growth in medium containing 10 μM Mg^2+^ (**Figure 1A**), as shown before (Li et al., 2001), while OsHKT2;4 did not (**Figure 1A**). These results indicated that although OsHKT2;4 had the Mg^2+^ transport activity similar to AtMGT10, it might have the kinetic property with lower affinity to Mg^2+^ in heterologous MM281 system.

To further verify the complementation of OsHKT2;4 for the growth of MM281 tested in the agar plates (**Figure 1A**), the bacteria were cultured in the liquid media containing 0.1, 0.5, 1, or 10 mM Mg^2+^, and their growth curves were established within 24 h after cultured. As shown in **Figure 1B**, the strain expressing AtMGT10 grew the most rapidly at these Mg^2+^ concentrations, supporting that AtMGT10 is a high-affinity Mg^2+^ transporter. In addition, MM281 expressing OsHKT2;4 displayed faster growth than those with empty vector pTrc99A under the conditions in which the Mg^2+^ concentration was 1 or 10 mM. By contrast, in the presence of 0.1 or 0.5 mM Mg^2+^, the strains expressing OsHKT2;4 displayed the similar rate of growth to the strains expressing empty vector pTrc99A. These results were consistent with the ones observed on the agar plates, and demonstrated that OsHKT2;4 might mediate low-affinity Mg^2+^ uptake *in vivo*.

### Mg^2+^-Dependent Currents Generated by OsHKT2;4 Expressing *X. laevis* Oocytes under High-Mg^2+^ Conditions

**FIGURE 2:**
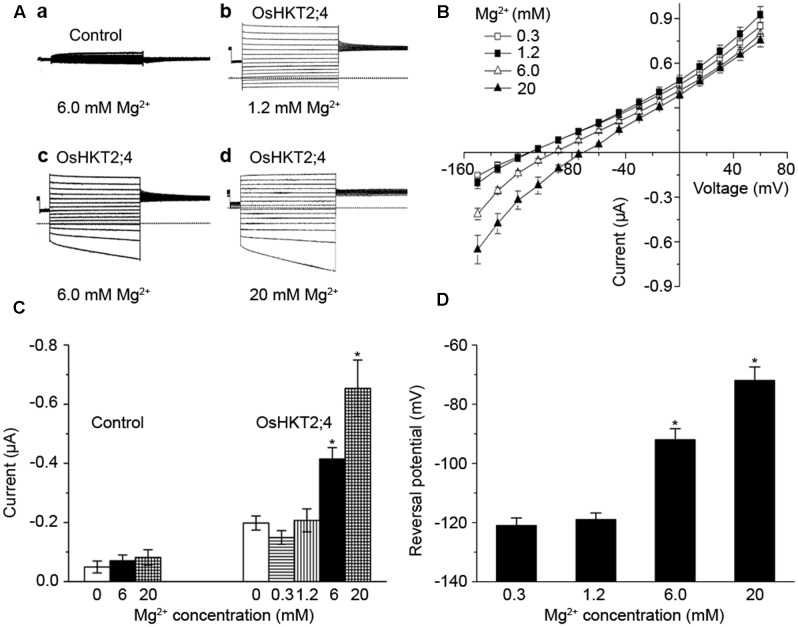
The oocytes expressing OsHKT2;4 produced Mg^2+^ currents under the high-Mg^2+^ conditions. **(A)** The typical current traces generated from the oocytes injected with water perfused with **(a)** 6 mM Mg^2+^ (Control) and from the oocytes expressing OsHKT2;4 (OsHKT2;4) perfused with **(b)** 1.2 mM, **(c)** 6 mM or **(d)** 20 mM Mg^2+^. Dotted lines represent the zero current level. **(B)** The current-voltage relationships deduced from the oocytes expressing OsHKT2;4 perfused with 0.3, 1.2, 6 or 20 mM Mg^2+^. Summarized current data are from 8 cells/condition. **(C)** The current amplitudes at –150 mV recorded from the oocytes injected with water (Control) and oocytes expressing OsHKT2;4 (OsHKT2;4) perfused with different Mg^2+^ concentrations. **(D)** Reversal potentials of currents generated from the oocytes expressing OsHKT2;4 in the presence of various concentrations of Mg^2+^ as indicated in the figure. Data in **Figure 2** are presented as representative recordings or as mean ± SE of *n* (*n* = 6) observations with three repetitions, in which *n* is the number of samples. Asterisks indicate statistically significant differences compared with data from oocytes expressing OsHKT2;4 perfused with 1.2 mM Mg^2+^ (Unpaired student’s *t*-test, ^∗^*P* < 0.05).

To further assess the transporting properties of OsHKT2;4 under different Mg^2+^ concentrations, two-electrode voltage-clamp experiment using *X. laevis* oocytes was performed. OsHKT2;4-dependent currents were recorded from the oocytes injected with OsHKT2;4 cRNA or the oocytes injected with water perfused with different Mg^2+^ concentrations. The oocytes injected with water produced small endogenous currents in perfusion medium with 6 mM Mg^2+^ (**Figures 2A-a**). In contrast, *OsHKT2;4*-expressing oocytes generated the larger currents in the solutions containing 1.2, 6, and 20 mM Mg^2+^ (**Figures 2A-b–d**). The current-voltage relationship displayed the currents from *OsHKT2;4*-expressing oocytes perfused with 6 or 20 mM Mg^2+^ were significantly larger than those from *OsHKT2;4*-expressing oocytes perfused with 0.3 or 1.2 mM Mg^2+^. It was noteworthy that the currents from OsHKT2;4-expressing oocytes perfused with 0.3 mM Mg^2+^ were similar to those with 1.2 mM Mg^2+^, implying they might not be Mg^2+^ sensitive under low-Mg^2+^ conditions (**Figure 2B**).

To test this possibility, we compared the amplitude and reversal potential of the currents generated from the oocytes perfused with 0, 0.3, 1.2, 6, or 20 mM Mg^2+^. The currents generated from the oocytes expressing OsHKT2;4 perfused with 0.3 or 1.2 mM Mg^2+^ displayed the similar levels of the amplitudes and reversal potentials, even were similar to those without Mg^2+^ (**Figures 2C,D**). These Mg^2+^ insensitive currents were larger than the currents from the oocytes injected with water (**Figure 2C**), and thus they may result from other ions, such as Na^+^ or K^+^ currents generated by OsHKT2;4, as suggested by the previous studies (Lan et al., 2010; Horie et al., 2011). By contrast, in the presence of 6 or 20 mM Mg^2+^, the oocytes expressing OsHKT2;4 produced the currents with larger amplitudes and less negative reversal potentials compared with the others (**Figures 2C,D**). Thus, these results further supported the hypothesis that OsHKT2;4 exhibits permeability for Mg^2+^ only under the conditions containing high-Mg^2+^ concentrations.

### Overexpression of OsHKT2;4 Enhanced the Sensitivity of *atmgt6* to High Mg^2+^ But Not to Low Mg^2+^

**FIGURE 3:**
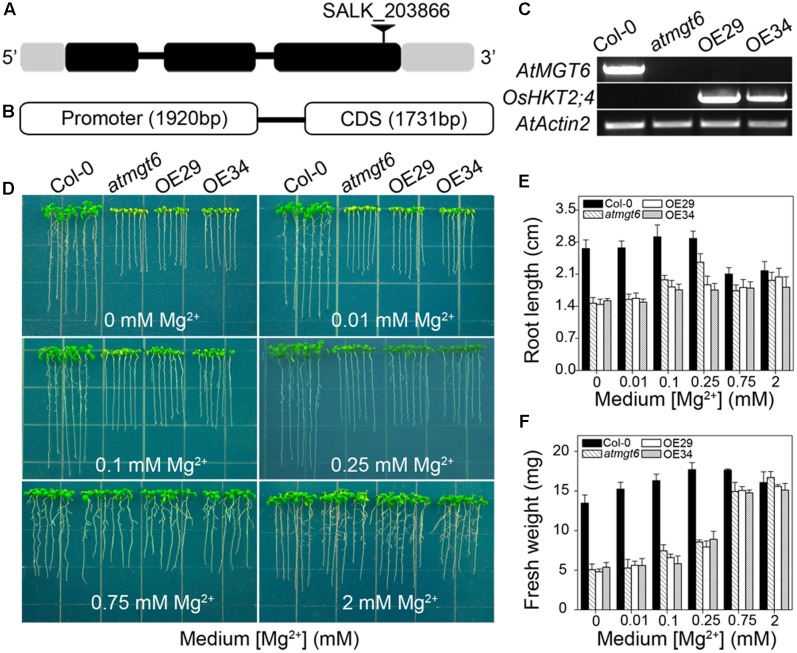
Genetic characterization and phenotypic analysis of *atmgt6* mutant lines and their OsHKT2;4 overexpressed lines in low-Mg^2+^ conditions. **(A)** Scheme of *AtMGT6* gene structure and position of the T-DNA insertion of SALK_203866. The gray boxes indicate 5′ and 3′ untranslated regions, and black boxes and lines indicate exons and introns, respectively. The T-DNA insertion is shown as the triangle above the gene diagram. **(B)** Gene fragment of *OsHKT2;4*, including its promoter and CDS region that was introduced into *atmgt6* lines. **(C)** Semi-quantitative mRNA levels of *AtMGT6* and *OsHKT2;4* by RT-PCR analysis in wild type (Col-0), *AtMGT6* knockout mutant (*atmgt6*), and two *atmgt6* lines overexpressing OsHKT2;4 (OE29 and OE34). *AtActin2* was used as the internal standard. **(D)** The growth of Col-0, *atmgt6*, OE29, and OE34 under the Mg^2+^-deficient conditions. After planted in half-strength Murashige and Skoog (1/2 MS) medium for 3 days, Col-0, *atmgt6*, OE29, and OE34 were transferred to one-sixth-strength MS (1/6 MS) medium containing 0, 0.01, 0.1, 0.25, 0.75, and 2 mM Mg^2+^ in total, and were photographed after growing for 7 days. Quantitative analyses of primary root length **(E)** and whole-plant fresh weight **(F)** of Col-0, *atmgt6*, OE29, and OE34 under the Mg^2+^-deficient conditions described in **(D)**. Six independent 10-day-old seedlings of each genotype were gathered as one biological repeat for root length and fresh weight measurement. Data are represented as the mean ± SD, *n* = 3, in which *n* is the number of biological repeat.

We have shown previously that AtMGT6, a Mg^2+^ deficiency-induced Mg^2+^ transporter, mediates directly Mg^2+^ uptake in roots and is required for plant adaptation to low-Mg^2+^ environment (Mao et al., 2014). An independent study reported ethyl methanesulfonate (EMS)-mutagenized *AtMGT6*, or named as *AtMRS2-4*, caused plant growth defects under both low and high-Mg^2+^ conditions (Oda et al., 2016). Considering the critical role of AtMGT6 in Mg^2+^ acquisition, we suggested the activity of Mg^2+^ transport conducted by OsHKT2;4 might be covered by this transporter, and thus generated transgenic *OsHKT2;4 Arabidopsis* lines with the disruption of *AtMGT6* to examine the potential relevance of OsHKT2;4 to Mg^2+^ responses *in planta*. We used an *Arabidopsis* T-DNA insertion line (SALK_203866), in which T-DNA was inserted into the third exon of *AtMGT6* gene (**Figure 3A**). Transcript of *AtMGT6* in the line SALK_203866 was not detected by RT-PCR (**Figure 3C**), indicating that the T-DNA insertion line was a knockout allele, and referred to as *atmgt6* line hereafter. We then expressed the coding region of *OsHKT2;4* into *atmgt6* line driven by its native promoter (**Figure 3B**). The transformants were screened by hygromycin, and were further analyzed for the expression levels of *OsHKT2;4* by RT-PCR. We selected two of them as the representative transgenic lines due to their relatively high *OsHKT2;4* expression levels, and referred to as OE29 and OE34, respectively, to perform subsequent experiments (**Figure 3C**).

We examined the growth of the Col-0, *atmgt6*, and transgenic lines OE29 and OE34 in Mg^2+^-depleted medium supplemented with various contents of Mg^2+^ as indicated in **Figure 3D**. The mutant *atmgt6* exhibited growth defects in the medium containing 0, 0.01, 0.1, or 0.25 mM Mg^2+^, and had lower fresh weight and shorter roots than those of the Col-0 plants, while the growth retardation could be rescued in the Mg^2+^-sufficient medium (2 mM Mg^2+^) (**Figure 3D**), consistent with the idea that *AtMGT6* confers low-Mg^2+^ tolerance for *Arabidopsis* (Mao et al., 2014; Oda et al., 2016). However, OsHKT2;4 overexpression could not rescue the growth deficiency of *atmgt6* in low-Mg^2+^ conditions as expected, as transgenic lines OE29 and OE34 displayed the similar growth phenotype to *atmgt6* with no significant differences on fresh weight and root length under these tested conditions (**Figures 3D–F**). These results suggested that OsHKT2;4 might not function in low-Mg^2+^ conditions *in planta*.

**FIGURE 4:**
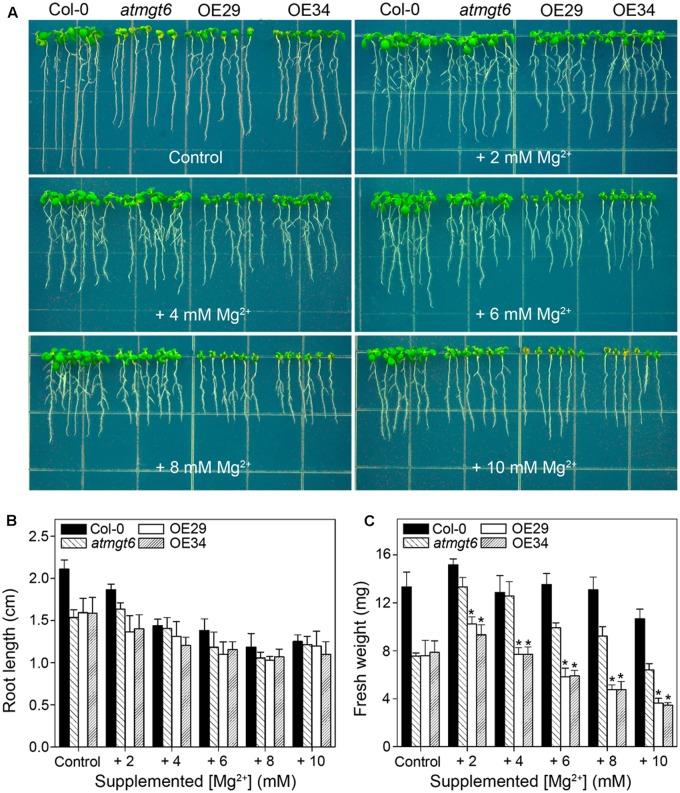
Growth phenotype of *atmgt6* and transgenic *OsHKT2;4*-overexpression *atmgt6* lines in high-Mg^2+^ conditions. **(A)** The growth of Col-0, *atmgt6*, OE29, and OE34 under the Mg^2+^-abundant conditions. After planted in half-strength MS (1/2 MS) medium for 3 days, Col-0, *atmgt6*, OE29, and OE34 were transferred to one-sixth-strength MS (1/6 MS, referred to as “Control” in the figure) containing a basal 0.25 mM Mg^2+^ and 1/6 MS medium supplemented with extra 2, 4, 6, 8, and 10 mM Mg^2+^. Plants were photographed after growing for another 7 days. Quantitative analyses of primary root length **(B)** and whole-plant fresh weight **(C)** of Col-0, *atmgt6*, OE29, and OE34 under the Mg^2+^-abundant conditions described in **(A)**. Six independent 10-day-old seedlings of each genotype were gathered as one biological repeat for root length and fresh weight measurement. Data are represented as the mean ± SD, *n* = 3, in which *n* is the number of biological repeat. Asterisks indicate statistically significant differences compared with *atmgt6* (Student’s *t*-test, ^∗^*P* < 0.05).

As shown in the experiments *in vitro*, OsHKT2;4 exhibiting Mg^2+^ transport activity in both heterologous MM281 system and *X. laevis* oocytes happened only at high external Mg^2+^ concentrations (**Figures 1**, **2**). Therefore, we presumed that OsHKT2;4 might mediate Mg^2+^ transport when plants were cultivated under the Mg^2+^ abundant conditions, though it was unable to function in low-Mg^2+^ conditions *in planta* (**Figures 3D–F**). To conduct assessment of the sensitivity to high-Mg^2+^ condition of OsHKT2;4, we used 1/6 MS medium supplemented with several concentrations of Mg^2+^ (2, 4, 6, 8, and 10 mM) for growth assays. After growing on 1/6 MS for 2 weeks, both the root length and fresh weight of transgenic lines OE29 and OE34 were comparable to those of *atmgt6* under the normal condition. However, addition of 2 mM Mg^2+^ resulted in growth arrest of OE29 and OE34 compared with *atmgt6*. Further increases of extra Mg^2+^ (up to 10 mM) demonstrated a consistent dosage-dependent inhibitory manner (**Figure 4A**). Quantitative analysis of root length (**Figure 4B**) and fresh weight (**Figure 4C**) indicated that, compared with Col-0 and *atmgt6*, the aerial parts of OE29 and OE34 exhibited a more severe growth retardation in Mg^2+^-abundant conditions, while their root length were not altered. Taken together, these results demonstrated that OsHKT2;4 results in Mg toxicity on aerial tissues in high-Mg^2+^ conditions in *Arabidopsis*, supporting the idea of low-affinity Mg^2+^ uptake of OsHKT2;4.

To examine whether the high-Mg^2+^ toxic phenotype is a consequence of the ectopic expression of OsHKT2;4 in OE lines, we conducted an RT-PCR analysis to verify the expression of OsHKT2;4 under a high-Mg^2+^ condition supplemented with 6 mM Mg^2+^. As demonstrated in Supplementary Figure 5, OsHKT2;4 was mainly expressed in shoot tissues under normal growth conditions, consistent with the previous report in rice (Lan et al., 2010). Moreover, the expression of *OsHKT2;4* in the shoots was not significantly induced by 6 mM Mg^2+^, and even decreased after 24 h′ treatment with 6 mM Mg^2+^. However, expression of *OsHKT2;4* in the roots was dramatically induced after 4 h′ treatment with 6 mM Mg^2+^, and became even stronger after 24 h of this treatment (Supplementary Figure 5). The oppose effect on *OsHKT2;4* expression roots and shoots upon high Mg^2+^ suggested a disturbance on Mg^2+^ balance between roots and shoots under the high-Mg^2+^ conditions.

### Overexpression of OsHKT2;4 Affected Mg^2+^ Homeostasis in the *atmgt6* Lines

**FIGURE 5:**
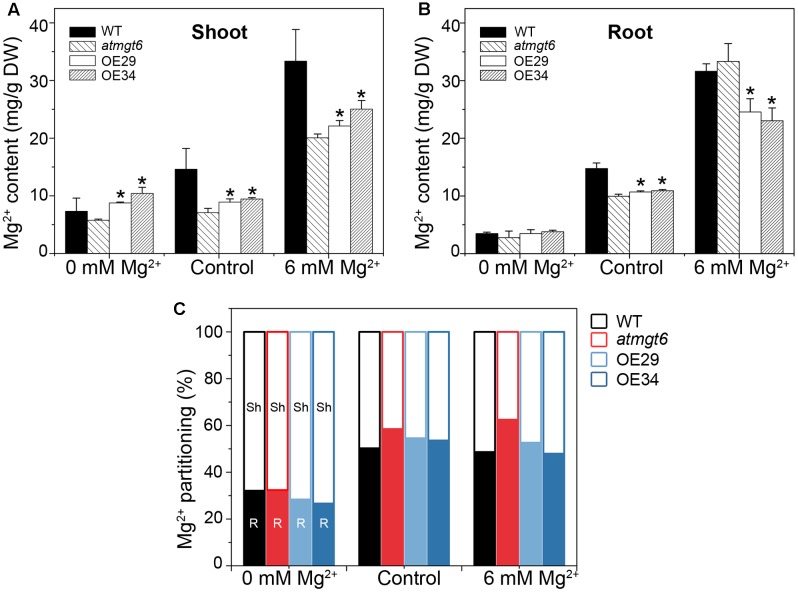
Mg^2+^ content and its partitioning in shoots and roots of *atmgt6* and transgenic *OsHKT2;4*-overexpression *atmgt6* lines. Inductively Coupled Plasma-Mass Spectrometry (ICP-MS) analysis of Mg^2+^ contents in shoots **(A)** and roots **(B)** of Col-0, *atmgt6* and two transgenic *OsHKT2;4*-overexpression *atmgt6* lines, OE29 and OE34. After planted in the hydroponic medium containing 0.25 mM Mg^2+^ for 2 weeks, Col-0, *atmgt6*, OE29, and OE34 were transferred to the hydroponic medium containing 0, 0.25 (Control), or 6 mM Mg^2+^, and were harvested for elemental analysis of roots and shoots after growing for 2 days. Data are represented as the mean ± SD, *n* = 3. Asterisks indicate statistically significant differences compared with *atmgt6* (Student’s *t*-test, ^∗^*P* < 0.05). **(C)** Altered Mg^2+^ partitioning between shoot (Sh) and root (R) in Col-0, *atmgt6*, OE29, and OE34. Values are deduced from **(A,B)**.

To probe the reason responsible for the increased sensitivity to high external Mg^2+^ in the OE plants, Mg^2+^ concentration of the *atmgt6* and OsHKT2;4 overexpression lines was determined using ICP-MS. Plants were grown hydroponically for 2 weeks and then transferred to a fresh hydroponic medium containing 0, 0.25 (referred to as “Control” in **Figure 5**), and 6 mM Mg^2+^ for another 2 days before the roots and shoots were harvested, respectively, for analysis. As shown in **Figure 5A**, Mg^2+^ content was consistently higher in the OE lines than *atmgt6* in shoot tissues when plants grown in all Mg^2+^ regimes tested (0, 0.25, and 6 mM). In analysis of Mg^2+^ content of root tissues among different plants, although they exhibited similar and incremental Mg^2+^ content in the Mg^2+^-deficient and normal medium, the OE lines contained ∼30% less Mg^2+^ compared with the *atmgt6* plants in 6 mM Mg^2+^ condition (**Figure 5B**). These results indicated an altered Mg^2+^ distribution ratio in shoot and root. We thus analyzed the Mg^2+^ partitioning between shoots and roots in *atmgt6* and OE lines, and noticed that as the Mg^2+^ level elevated, more Mg^2+^ sequestered in root tissues than in low-Mg^2+^ condition. In medium containing 6 mM Mg^2+^, the shoots of *atmgt6* accumulated ∼37%, while the OE lines accumulated over ∼50% of the total Mg^2+^ enclosed in plants (**Figure 5C**), further confirming the critical role of OsHKT2;4 in the Mg^2+^ allocation between shoots and roots.

### Increased Sensitivity of *atmgt6* Lines Expressing OsHKT2;4 to Excess Mg^2+^ Was Alleviated by Adding Ca^2+^

Due to the similar physical properties, Ca^2+^ and Mg^2+^ compete for the same sites of substrates (Yermiyahu et al., 1994), and the balance of Ca^2+^ and Mg^2+^ is an important factor for plant growth. Previously, evidence was presented that OsHKT2;4 acts as a channel for the transport of both Ca^2+^ and Mg^2+^ (Lan et al., 2010; Horie et al., 2011). To examine whether Ca^2+^ affects the high Mg^2+^-sensitive phenotype in OsHKT2;4 overexpressing lines, we assessed the growth of Col-0, *atmgt6*, *atmgt6* overexpressing OsHKT2;4 lines (OE29 and OE34) on Mg^2+^-abundant 1/6 MS medium supplemented with different concentrations of excess Ca^2+^.

**FIGURE 6:**
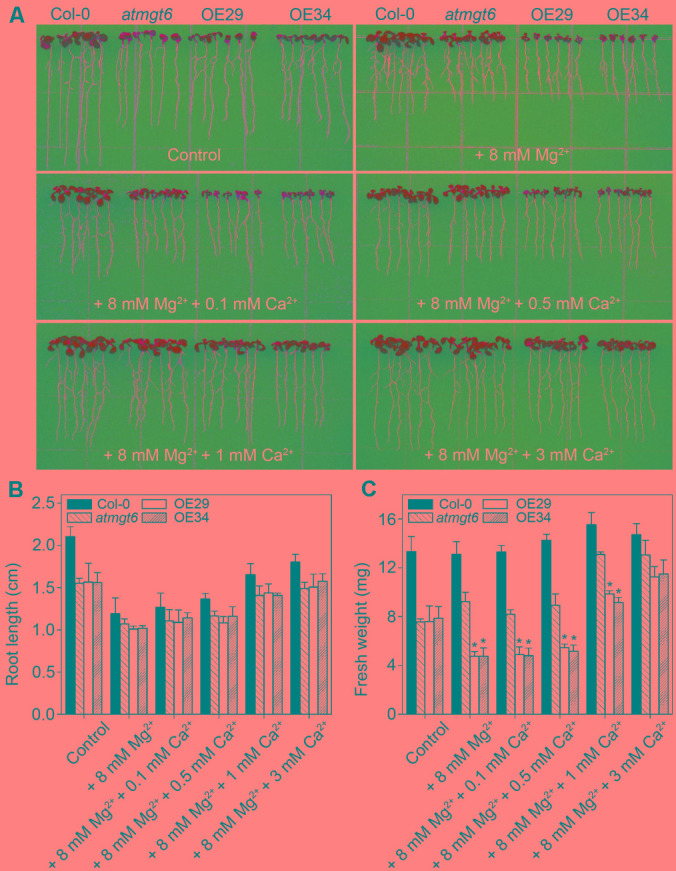
Effects of high Ca^2+^ additions on the growth of *atmgt6* and transgenic *OsHKT2;4*-overexpression *atmgt6* lines in the Mg^2+^-abundant medium. **(A)** The growth of Col-0, *atmgt6* and two transgenic *OsHKT2;4* overexpression *atmgt6* lines (OE29 and OE34) in 1/6 MS (Control) and the Mg^2+^-abundant medium (1/6 MS with extra 8 mM Mg^2+^, referred to as “+8 mM Mg^2+^” in the figure) containing different extra Ca^2+^ concentrations. After planted in 1/2 MS medium for 3 days, Col-0, *atmgt6*, OE29, and OE34 were transferred to the 1/6 MS medium (containing a basal 0.25 mM Mg^2+^ and 0.5 mM Ca^2+^), or the Mg^2+^-abundant medium with 0, 0.1, 0.5, 1, or 3 mM extra Ca^2+^. Plants were photographed after growing for another 7 days. Quantitative analyses of primary root length **(B)** and whole-plant fresh weight **(C)** of Col-0, *atmgt6*, OE29, and OE34 under the conditions described in **(A)**. Six independent 10-day-old seedlings of each genotype were gathered as one biological repeat for root length and fresh weight measurement. Data are represented as the mean ± SD, *n* = 3, in which *n* is the number of biological repeat. Asterisks indicate statistically significant differences compared with *atmgt6* (Student’s *t*-test, ^∗^*P* < 0.05).

In normal 1/6 MS medium, OE29 and OE34 exhibited the similar growth as *atmgt6*. As the concentration of external Mg^2+^ increased, OE29 and OE34 started to show a more severe growth arrest than *atmgt6* (**Figure 4A**). However, increasing Ca^2+^ improved the plant growth (**Figure 6A**) of all genotypes, and the improvement was more obvious in the OE29 and OE34 lines (Supplementary Figure 2). For example, fresh weight of OE lines was ∼50% of that in *atmgt6* in 1/6 MS medium with extra 8 mM Mg^2+^ (“basal medium” in **Figure 6**), however, when 1 mM Ca^2+^ was added to this basal medium, the fresh weight of OE lines was restored and reached to ∼80% to that in *atmgt6*. Moreover, when 3 mM Ca^2+^ were added to the basal medium, both root length and fresh weight of OE29 and OE34 were recovered to almost an identical level with that of *atmgt6* (**Figures 6B,C** and Supplementary Figure 2), supporting the notion of Ca^2+^-Mg^2+^ antagonism.

**FIGURE 7:**
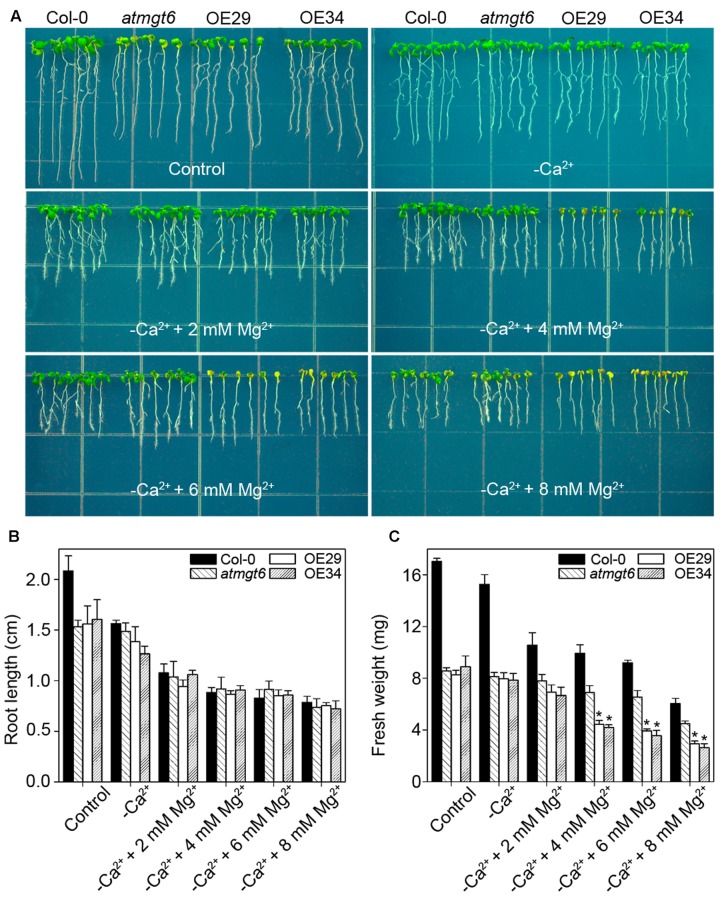
Effects of the Ca^2+^ depletion on the growth of *atmgt6* and transgenic *OsHKT2;4*-overexpression *atmgt6* lines in the Mg^2+^-abundant medium. **(A)** The growth of Col-0, *atmgt6* and transgenic two *OsHKT2;4* overexpression *atmgt6* lines (OE29 and OE34) in 1/6 MS (Control) and the 1/6 MS medium depleted of Ca^2+^ (–Ca^2+^). After planted in 1/2 MS medium for 3 days, Col-0, *atmgt6*, OE29, and OE34 were transferred to the 1/6 MS medium (containing a basal 0.25 mM Mg^2+^ and 0.5 mM Ca^2+^), or the 1/6 MS medium depleted of Ca^2+^ and supplemented with extra 2, 4, 6, or 8 mM Mg^2+^. Plants were photographed after growing for another 7 days. Quantitative analyses of primary root length **(B)** and whole-plant fresh weight **(C)** of Col-0, *atmgt6*, OE29, and OE34 under the conditions described in **(A)**. Six independent 10-day-old seedlings of each genotype were gathered as one biological repeat for root length and fresh weight measurement. Data are represented as the mean ± SD, *n* = 3, in which *n* is the number of biological repeat. Asterisks indicate statistically significant differences compared with *atmgt6* (Student’s *t*-test, ^∗^*P* < 0.05).

It has been reported that low Ca^2+^ in the medium triggered the increase of Mg^2+^ concentration, mimicking high-Mg^2+^ conditions (Rios et al., 2012). To analyze further whether Ca^2+^ deficiency is also responsible for the phenotype induced by high Mg^2+^, we thus tested the sensitivity among different plants to low-Ca^2+^ conditions. In Ca^2+^-depleted 1/6 MS medium (“basal medium” in **Figure 7**), similar phenotype was observed in *atmgt6* and OE lines, suggesting that OsHKT2;4 is not responsible for Ca^2+^ deficiency. On the contrary, as the increasing content of extra Mg^2+^ (2, 4, 6, and 8 mM) was added to the basal medium, differences of fresh weight between *atmgt6* and OE lines started to occur (**Figure 7** and Supplementary Figure 3). These results demonstrated that high Mg^2+^, rather than Ca^2+^ deficiency, is the primary factor that caused growth defects in the OE lines than *atmgt6*.

## Discussion

Mg^2+^ is an essential macronutrient for plant growth, development and reproductive success (Li et al., 2001; Hermans et al., 2013; Mao et al., 2014), while it could be detrimental at high concentrations (Visscher et al., 2010). Plants possess specific Mg^2+^ transport systems that can function under a wide range of concentrations to secure intracellular Mg^2+^ homeostasis. Despite several transporters have been shown to function in Mg^2+^ uptake and distribution in *Arabidopsis*, including the AtMGT/AtMRS2-type transporters (Li et al., 2001) and Mg^2+^/H^+^ antiporter AtMHX (Shaul et al., 1999), little is known about the transporters responsible for Mg^2+^ homeostasis in rice. OsHKT2;4 has been reported to function as a non-selective transporter for diverse cations, including Mg^2+^ and Ca^2+^. Our study here provided further evidence that OsHKT2;4 exhibits characteristics of low-affinity transport of Mg^2+^, and plays a key role in Mg^2+^ homeostasis for plant’s adaptation to high-Mg^2+^ conditions.

Rice contains up to nine *HKT* genes (depending on variety), and OsHKT2;4 is the member of class II HKTs with the conserved Gly residues at the four P-loop filter positions (Mäser et al., 2002b). OsHKT2;4 is localized at the plasma membrane of rice cells, and its exogenous expression caused *X. laevis* oocytes to produce large currents when the extracellular Mg^2+^ concentrations were at the range of millimolar levels (Lan et al., 2010; Horie et al., 2011). *Triticum aestivum* HKT2;1 (TaHKT2;1) was also found to result in robust Mg^2+^ permeability of the oocytes, although to a lesser degree (Horie et al., 2011). OsHKT2;4-mediated currents exhibited the shifts to positive reversal potentials upon increased Mg^2+^ concentration from 5 to 50 mM (Horie et al., 2011). The present study further analyzed the capability of Mg^2+^-uptake of OsHKT2;4 in three systems, the oocytes, bacteria, and *Arabidopsis* under the conditions containing high-Mg^2+^ concentrations. The current amplitudes and reversal potentials in the oocytes expressing OsHKT2;4 were not changed when the extracellular Mg^2+^ concentration was less than 1.2 mM, until its concentration reached 6 mM (**Figure 2**). Similarly, the expression of OsHKT2;4 rescued growth defects of MM281 bacteria cells that are deficient in Mg^2+^ uptake in the presence of relatively high-Mg^2+^ concentration, but the rescuing effect was much less than MGT10, the high-affinity Mg^2+^ transporter (**Figure 1**). Complementary to the observations in the oocytes and bacteria, the phenotype relating to Mg^2+^ stress in transgenic OsHKT2;4-overexpressed *atmgt6* lines happened at the Mg^2+^-abundant (**Figure 4**), but not at Mg^2+^-deficient conditions (**Figure 4**). Taken together, our findings showed that OsHKT2;4 has a distinct low-affinity Mg^2+^ transportation, and confirm Mg^2+^ permeability of OsHKT2;4 as reported (Lan et al., 2010; Horie et al., 2011). It is worth mentioning that OsHKT2;4 was reported to be impermeable to Mg^2+^ when it was expressed in the oocytes by an independent study (Sassi et al., 2012). Due to the genetic diversity in rice during evolution and amino acid variation of HKTs among *Oryza* accessions (Horie et al., 2001; Ren et al., 2005), the differences might result from the sources of OsHKT2;4 from different rice varieties. In the previous studies (Lan et al., 2010; Horie et al., 2011) and the present study, genetically tractable rice (*O. sativa*; background Nipponbare) was used.

Mg^2+^ is taken up from the soil by the plant root system, which is likely to be mediated by AtMGT6/MRS2-4. AtMGT6/MRS2-4 is located in the plasma membrane or the endoplasmic reticulum and highly expressed in the root epidermal cells, and its disruption resulted in growth retardation of *Arabidopsis* under the low-Mg^2+^ condition (Mao et al., 2014; Oda et al., 2016). OsHKT2;4 might not be a key factor for roots to uptake Mg^2+^ from the low-Mg^2+^ environment as its overexpression did not cause the changed growth phenotype of transgenic lines (**Figure 3**), fitting the idea of its low-affinity Mg^2+^ transportation. After satisfying the needs of the roots, the rest of the Mg^2+^ will be transported to the shoot through the process involving AtMGT6/MRS2-4 activity (Oda et al., 2016). However, plants will display Mg^2+^ toxicity symptom when Mg^2+^ is over accumulated in the shoot. To deal with this toxicity, plants might restrain the Mg^2+^ distribution in shoot or sequester the excess intracellular Mg^2+^ into the vacuoles (Hermans et al., 2013; Tang et al., 2015). The OE lines had higher Mg^2+^ content in shoot compared with *atmgt6* under both low-Mg^2+^ and normal conditions (**Figure 5**), indicating the expression level of *OsHKT2;4* was high enough to drive transportation of Mg^2+^ from root to shoot. However, once the expression level of *OsHKT2;4* was further enhanced under high-Mg^2+^ conditions (Supplementary Figure 5), the Mg^2+^ transportation to shoot was also strengthened, thus leading to an increased Mg^2+^ distribution ratio of shoot to root and enhanced sensitivities of OE lines to high-Mg^2+^ conditions. Therefore, we suggested that OsHKT2;4 plays a key role in Mg^2+^ homeostasis and might control the Mg^2+^ translocation between roots and shoots under the high-Mg^2+^ conditions.

Although roles of Ca^2+^ and Mg^2+^ are distinct in diverse physiological and biochemical processes, they may play an antagonistic function in plants. (Tang and Luan, 2017). For example, growth retardations induced by individual knockouts of genes in AtMRS2/AtMGT family under low Mg^2+^ could be ameliorated when Ca^2+^ concentrations were concomitantly lowered (Lenz et al., 2013). Mutation of *AtCAX1*, which encodes a vacuolar Ca^2+^/H^+^ exchanger, resulted in reduction of Ca^2+^ in the vacuole, thus leading to more Ca^2+^ retaining in the cytosol to counteract with excess Mg^2+^ (Cheng et al., 2003; Bradshaw, 2005). Consistently, our study demonstrated that addition of Ca^2+^ to the high-Mg^2+^ medium could partially rescue the Mg^2+^-induced growth defect of *atmgt6* and the OsHKT2;4-overexpressed lines to a wild type level (**Figure 6**), which is also a supportive evidence for the antagonistic interaction between Ca^2+^ and Mg^2+^ *in planta*. However, Mg^2+^ currents through OsHKT2;4 in oocytes were not inhibited and their reversal potentials were not significantly shifted in the presence of 1.8 mM Ca^2+^ in the perfusion solution (Supplementary Figure 4), indicating that Ca^2+^ did not inhibit Mg^2+^ uptake in oocytes expressing OsHKT2;4. Thus, the effects of changes in Ca^2+^ additions on Mg^2+^ toxicity might result from the physiological antagonism between Ca^2+^ and Mg^2+^ *in planta* (Bradshaw, 2005; Tang et al., 2015), although further evidence is needed.

In our previous study, we found that OsHKT2;4 had the diverse expression pattern in rice plants, including leaves, stems and primary/lateral roots, and was highly expressed at xylem and phloem of epidermis (Lan et al., 2010). However, the homozygous lines of *Tos*-tagged *oshkt2;4* rice lines behaved similarly to wild-type plants (Lan et al., 2010; Horie et al., 2011), and contained similar content of cations, including Mg^2+^ and Ca^2+^ (Lan et al., 2010). The absence of a phenotypic change in these rice lines suggested that OsHKT2;4 is functionally redundant with other transporters. Indeed, we found OsHKT2;4 rendered *Arabidopsis* Mg^2+^ sensitivity when *atmgt6* was knockout (**Figure 4**). Rice (*O. sativa*; background Nipponbare) is predicted to have nine AtMGT orthologs based on the BLAST search (Gebert et al., 2009), and Os10g0545000, the closest one to AtMGT6, is also widely expressed in rice according to the microarray gene expression data collected by Genevestigator^1^ (Supplementary Figure 1). Elucidation of the functional relationships between MGT-type transporters and OsHKT2;4 will be a critical next step toward assessing their biological functions in rice.

## Author Contributions

CZ, BZ, HL, JG, and WL designed the study. CZ, HL, BZ, and WL performed experiments. CZ, HL, JW, BZ, WW, JG, and WL analyzed and interpreted the data. CZ, HL, BZ, and WL wrote the manuscript. CZ, BZ, HL, SL, JG, and WL revised the manuscript critically. All authors read and approved the final manuscript.

## Conflict of Interest Statement

The authors declare that the research was conducted in the absence of any commercial or financial relationships that could be construed as a potential conflict of interest.

## Footnotes

1 www.genevestigator.com
