# Supplementary material for: The Rice High-Affinity K+ Transporter OsHKT2;4 Mediates Mg2+ Homeostasis under High-Mg2+ Conditions in Transgenic Arabidopsis
Source: Front Plant Sci. 2017 Oct 24;8:1823. doi: 10.3389/fpls.2017.01823 (PMC5660728; doi:10.3389/fpls.2017.01823)
Supplement: Supplementary file 1 [file Table_1.DOCX]

| Primers | Sequences (5’ to 3’) | Purposes |
| --- | --- | --- |
| atmgt6-F | TGCCCTCCCAGCACATAATACATC | Mutant identificaion |
| atmgt6-R | GAGAATTTTAGGGTTTCGGAAAGTAAATCTG |  |
| AtActin2-F | TCCCTCAATCTCATCTTCTTCC | Mutant identificaion |
| AtActin2-R | GACCTGCCTCATCATACTCG |  |
| OsHKT2;4-F | ATGCCTATTCGGCTGCATATC | Mutant identificaion |
| OsHKT2;4-R | TCACCTAGTTCGTGCGCTTG |  |
| OsHKT2;4-FC | CGGAATTCATGCCTATTCGGCTGCATATCTTTGTCAG | Expression in MM281 |
| OsHKT2;4-RC | CGGGATCCTCACCTAGTTCGTGCGCTTGTGG |  |
| OsHKT2;4-OE-F | TCCCCCGGGGTGGCTGATAGAGGATTGATTT | Genomic complement |
| OsHKT2;4-OE-R | CGGAGCTCATAACATCATCACCTAGTTCGTG |  |
| OsHKT2;4-FP | TCCCCCGGG ATGCCTATTCGGCTGCATATC | Expression in oocytes |
| OsHKT2;4-RP | CGGGATCCTCACCTAGTTCGTGCGCTTGTGG |  |

**Supplementary Table 1 | Primers used in this study**
