# Supplementary material for: The Rice High-Affinity K+ Transporter OsHKT2;4 Mediates Mg2+ Homeostasis under High-Mg2+ Conditions in Transgenic Arabidopsis
Source: Front Plant Sci. 2017 Oct 24;8:1823. doi: 10.3389/fpls.2017.01823 (PMC5660728; doi:10.3389/fpls.2017.01823)
Supplement: Supplementary file 2 [file Data_Sheet_1.DOCX]

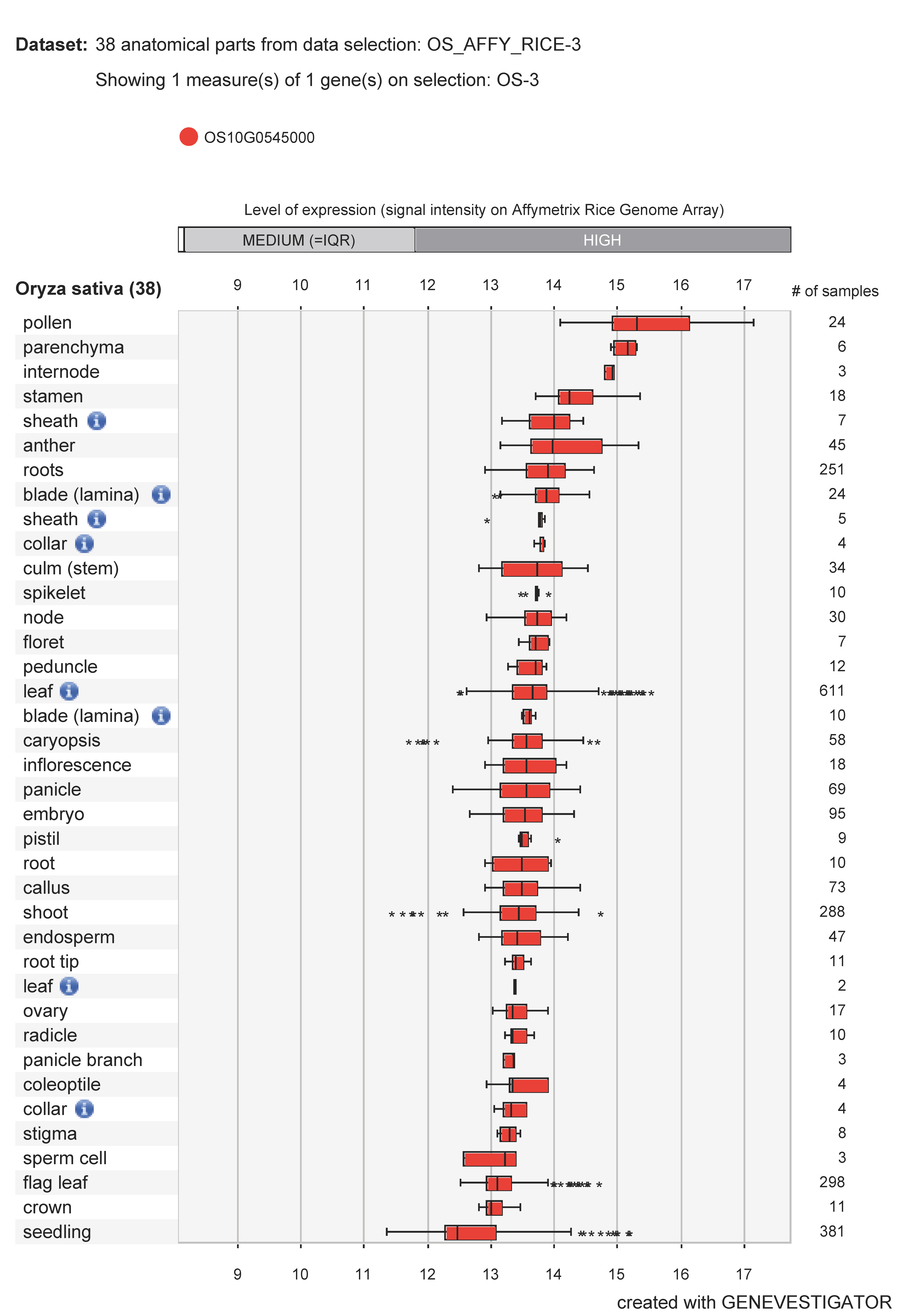


**Supplementary Figure 1 | Gene expression data collected by Genevestigator.**

**
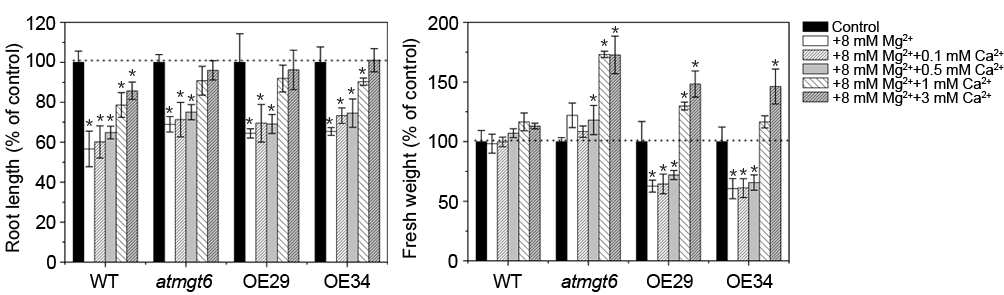
**

**Supplementary Figure 2 | Phenotype analysis of *atmgt6* and transgenic OsHKT2;4-overexpressing *atmgt6* lines in the Mg^2+^-abundant medium with different Ca^2+^ additions.**

Comparisons of the root length **(A)** and fresh weight **(B)** of Col-0, *atmgt6*, OE29 and OE34 across different conditions as indicated in Figure 6. Data were analyzed by two-way ANOVA followed by a Tukey’s post hoc test to identify mean differences between treatments and control conditions. Asterisks indicate statistically significant differences compared with control condition (**P* < 0.05). Statistical analyses were performed using the software GraphPad Prism.

**
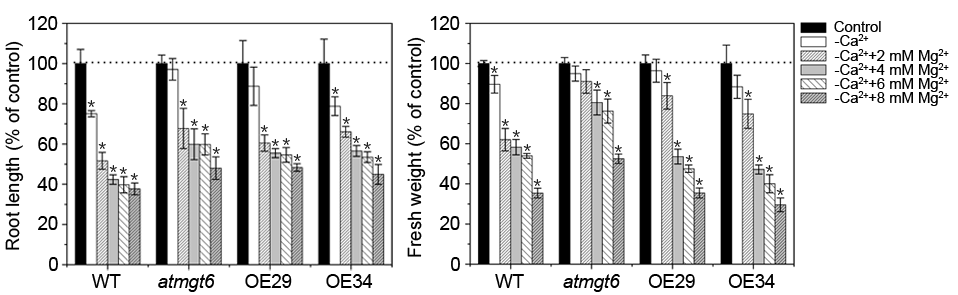
**

**Supplementary Figure 3 | Phenotype analysis of *atmgt6* and transgenic OsHKT2;4-overexpressing *atmgt6* lines in the Ca^2+^-depleted medium with different Mg^2+^ additions.**

Comparisons of the root length **(A)** and fresh weight **(B)** of Col-0, *atmgt6*, OE29 and OE34 across different conditions as indicated in Figure 7. Data were analyzed by two-way ANOVA followed by a Tukey’s post hoc test to identify mean differences between treatments and control conditions. Asterisks indicate statistically significant differences compared with control condition (**P* < 0.05). Statistical analyses were performed using the software GraphPad Prism.

**
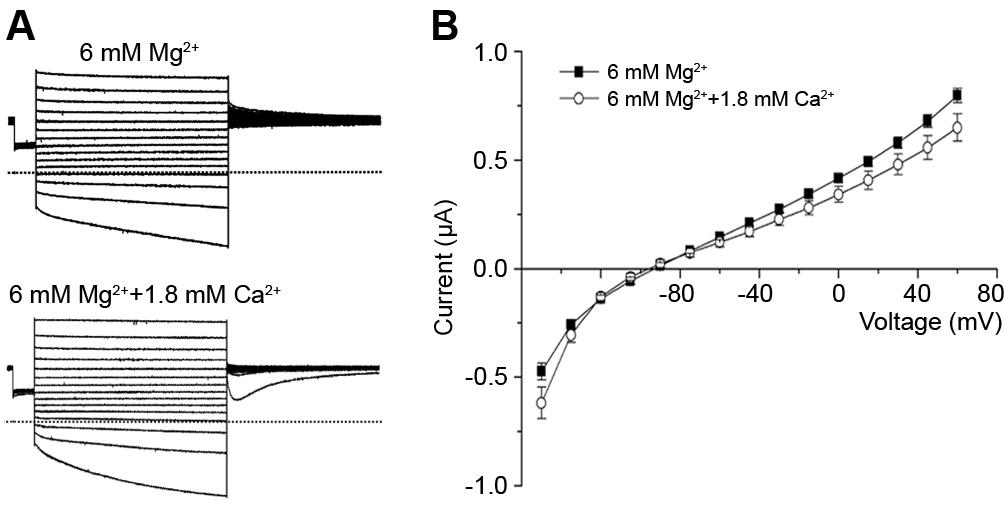
**

**Supplementary Figure 4 | Mg^2+^ currents produced by oocytes expressing OsHKT2;4 did not respond to increasing Ca^2+^. (A)** The current traces generated from oocytes expressing OsHKT2;4 perfused with 6 mM Mg^2+^ (6 mM Mg^2+^) or 6 mM Mg^2+^ and 1.8 mM Ca^2+^ (6 m M Mg^2+^+1.8 mM Ca^2+^). Dotted lines represent the zero current level. **(B)** The current-voltage relationships deduced from the oocytes expressing OsHKT2;4 perfused with 6 mM Mg^2+^ or 6 mM Mg^2+^+1.8 mM Ca^2+^. Data are presented as representative recordings or as mean ± SE of *n* (n=6) observations with three repetitions, in which *n* is the number of samples.

**
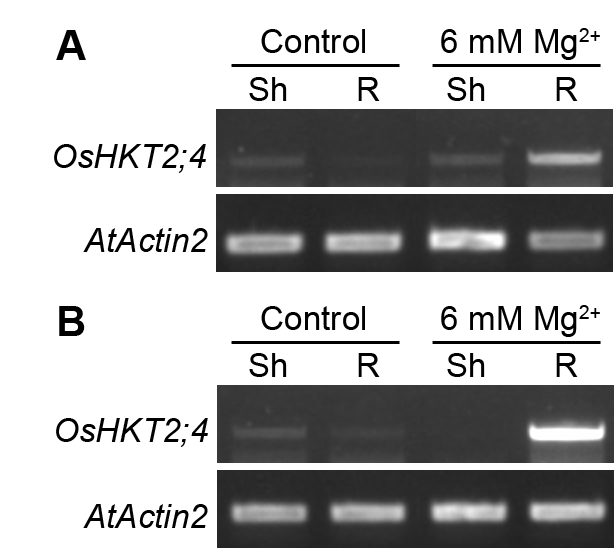
**

**Supplementary Figure 5 | Altered expression of OsHKT2;4 under high-Mg^2+^ conditions.** 2-week-old hydroponically cultured OE29 line was used, and was subjected to control condition (hydroponic 1/6 MS medium, referred to as “Control” in this figure) and high-Mg^2+^ condition (hydroponic 1/6 MS medium supplemented with 6 mM Mg^2+^, referred to as “6 mM Mg^2+^” in this figure), respectively. Shoots (Sh) and roots (R) were separately harvested after treated for **(A)** 4 hours and **(B)** 24 hours respectively for further RT-PCR analysis. *AtActin2* was used as the internal standard.
